# Case Report: Long-Term Survival With Anlotinib in a Patient With Advanced Undifferentiated Large-Cell Lung Cancer and Rare Tonsillar Metastasis

**DOI:** 10.3389/fonc.2021.680818

**Published:** 2021-06-24

**Authors:** Tianwei Xu, Chenchen Wei, Xiaoteng Zou, Binbin Lu, Zhaoxia Wang

**Affiliations:** Cancer Medical Center, The Second Affiliated Hospital of Nanjing Medical University, Nanjing, China

**Keywords:** undifferentiated large-cell lung cancer, antiangiogenic therapy, anlotinib, tonsillar metastasis, platelet-derived growth factor receptors (PDGFR)

## Abstract

Undifferentiated large-cell lung cancer is a rare type of non-small cell lung cancer (NSCLC) with a poor prognosis. It is insensitive to chemotherapy and easily develops drug resistance. Analysis of the Surveillance, Epidemiology, and End Results (SEER) database showed that patients with stage IV undifferentiated large-cell lung cancer had a median overall survival (OS) of only 4 months and that those who received chemotherapy had a median OS of only 5 months longer than those who did not. For the first time, we report a case of advanced large-cell undifferentiated lung cancer with rare tonsil metastasis. The patient developed resistance after 3 months of platinum-based systemic chemotherapy and local treatment. Antiangiogenic therapy has been continuously progressing and has shown certain efficacy in treating many malignant tumors, such as lung cancer. However, there are no relevant studies or case reports on antiangiogenic therapy in the treatment of undifferentiated large-cell lung cancer. Anlotinib, an orally delivered small-molecule antiangiogenic tyrosine kinase inhibitor (TKI), was administered to this patient after chemotherapy resistance occurred, and the outcome was assessed as continued stable disease (SD). As of the last follow-up evaluation, the progression-free survival (PFS) of the patient was 21.5 months, and the OS was 27.5 months. Retrospective immunohistochemical analysis showed that the patient was positive for one of the targets of anlotinib (PDGFR). In general, the findings in this case suggest that anlotinib may be an option with good efficacy for patients with large-cell undifferentiated lung cancer after chemotherapy resistance that may have good efficacy and also suggest that PDGFR may be the target underlying this effect.

## Introduction

Metastasis of tumors to the tonsil is extremely rare, with nearly 100 cases reported to date. The most common primary tumors are digestive tract tumors, renal cancer, and melanoma (1). Most metastatic tonsil tumors from lung cancer result from small cell lung cancer (2). No case of tonsil metastasis from undifferentiated large-cell lung cancer has been reported.

Antiangiogenic therapy has been recommended for the treatment of non-small cell lung cancer (NSCLC) (3), but there have been no clinical trials or case reports of antiangiogenic therapy for undifferentiated large-cell lung cancer. Anlotinib is an oral multitarget small-molecule tyrosine kinase inhibitor (TKI) (4). Its main targets include VEGFR and PDGFR. The Alter0303 study (5) showed that anlotinib as a third-line treatment can benefit advanced NSCLC patients with chemotherapy failure. However, patients with undifferentiated large-cell lung cancer were not included in this study; therefore, the efficacy of anlotinib in this group of patients is unknown. We present the first case of a patient with undifferentiated large-cell lung cancer with rare tonsillar metastases who achieved a long survival time with anlotinib treatment after chemotherapy failure. The following case is presented in accordance with the CARE reporting checklist.

## Case Presentation

**Figure 1:**
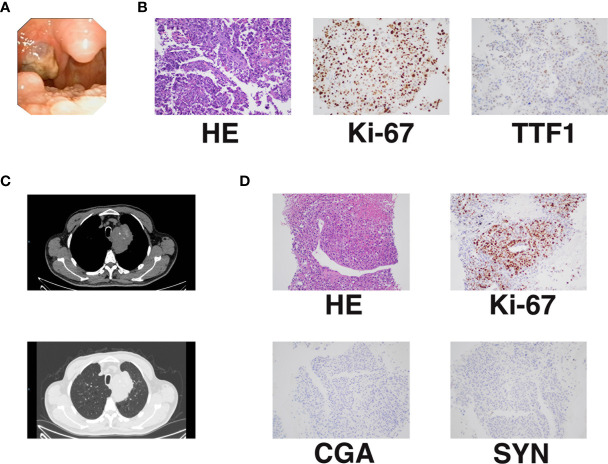
Initial diagnosis of advanced undifferentiated large-cell lung cancer with rare tonsillar metastasis. **(A)** Tonsillar metastasis. **(B)** Pathology and immunohistochemistry of tonsillar metastasis [KI-67 (40%+); TTF1 (±)]. **(C)** Primary lung lesion shown by chest CT. **(D)** Pathology and immunohistochemistry of the primary lung lesion [KI-67 (50%+); CGA (−; SYN (−)].

A 70-year-old man with a 20-year history of smoking presented to the otolaryngology department in January 2019 with a foreign body sensation in his throat. The patient occasionally had dry cough, without sputum, hemoptysis, chest tightness, dyspnea, and other symptoms. A physical examination showed that the trachea was centered, and there was no obvious abnormality in the respiratory sounds of either lung. No superficial lymph node enlargement was observed. The patient reported no family history of cancer. Laryngoscopy revealed a tonsil mass (**Figure 1A**). Chest computed tomography (CT) (January 16, 2019) showed a mass of 78 * 51 mm in the upper left mediastinum (**Figure 1C**). Cranial magnetic resonance imaging (MRI), abdominal CT, and whole body bone scan examinations showed no other metastatic lesions (**Supplementary Figure 1**). We performed puncture of the lung lesions and tonsillectomy for the tonsil lesions. Hematoxylin and eosin (HE) staining and Ki-67 immunohistochemistry of the tonsil lesion demonstrated malignancy. The TTF1 immunohistochemical results suggested that the lesion might originate from the lung (**Figure 1B**). HE staining and Ki-67 immunohistochemistry of lung tissue indicated large-cell carcinoma. The results of assessment of neuroendocrine-related immunohistochemical indexes, including CGA and SYN, were negative, indicating that the tumor may not have neuroendocrine function (**Figure 1D**). The patient was finally diagnosed with undifferentiated large-cell lung cancer of the left lung [T4NxM1b, American Joint Committee on Cancer (AJCC) 8^th^ edition]. Next generation sequencing (NGS, Geneseeq Technology Inc) found mutations in TP53, PIK3CA, and CD74 and no driver gene mutations that could be used for targeted therapy (**Table 1**).

**Table 1:** Mutations and their abundance detected by NGS (Geneseeq Technology Inc).

Genes	Mutations	Peripheral Blood	Cancer tissue
CD74	c.679C>G (p.L227V)	–	1.8%
PIK3CA	c.1637A>C (p.Q546P)	0.8%	8.4%
TP53	c.434T>A (p.L145Q)	1.7%	30.7%

**Figure 2:**
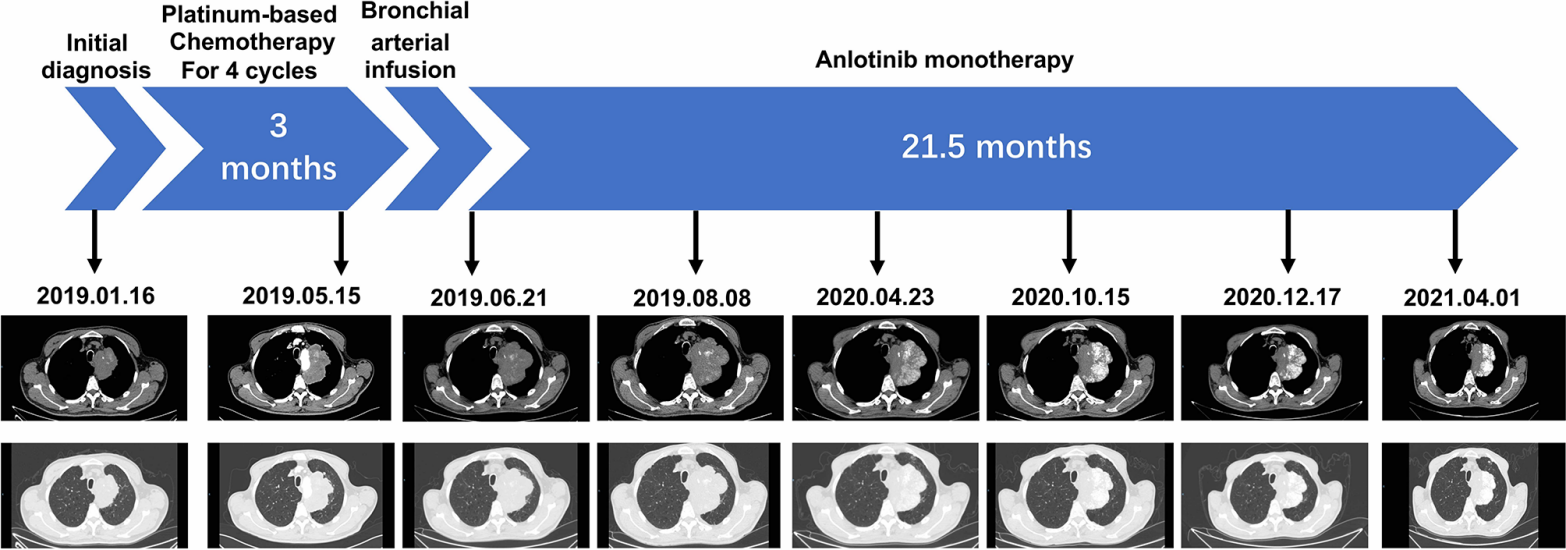
Graphic summary of the case.

As shown in **Figure 2**, from February 15, 2019, the patient received albumin-bound paclitaxel (200 mg on d 1 and 8) + carboplatin (450 mg on d 1) chemotherapy for four cycles. Chest CT (May 15, 2019) showed a 93 * 62 mm mass near the left upper mediastinum, and the efficacy was evaluated as progressive disease (PD). This result indicated that the patient had acquired resistance to chemotherapy, with a progression-free survival (PFS) time of 3 months for this chemotherapy regimen. A bronchial arterial infusion (cisplatin 40 mg + epirubicin 40 mg) was performed on May 22, 2019. Chest CT (June 6, 2019) showed a mass of 102 * 63 mm near the left upper mediastinum, which was evaluated as stable disease (SD). The patient refused further chemotherapy regimens. Immunotherapy was refused for financial reasons. Complying with the patient’s wishes, we administered oral antiangiogenic therapy with anlotinib (12 mg/d). Each cycle was defined as 2 weeks on treatment followed by 1 week off treatment. The patient was followed up at an outpatient clinic. The patient developed tolerable mild fatigue, diarrhea, and a small rash after initial use of anlotinib. These adverse reactions were rated as Level 1 (CTCAE 5.0). These adverse events resolved spontaneously without treatment after two cycles of anlotinib treatment. Chest CT (August 8, 2019) showed a 106 * 63 mm mass near the left upper mediastinum, and the efficacy was evaluated as SD. The patient continued oral treatment with anlotinib monotherapy. Chest CT (April 23, 2020) showed a 101 * 64 mm mass near the left upper mediastinum, and the efficacy was evaluated as SD. The patient continued to receive oral anlotinib monotherapy, and chest CT (October 15, 2020) showed a 93 * 52 mm mass near the left upper mediastinum. Chest CT scan (December 17, 2020.) showed a 93 * 52 mm mass near the left upper mediastinum. The last follow-up chest CT scan (April 1, 2021) showed an 83 * 52 mm mass near the left upper mediastinum, and the efficacy was evaluated as SD. At the last follow-up, the patient’s PFS and overall survival (OS) were 21.5 and 27.5 months, respectively, after anlotinib treatment. A re-examination of head and neck CT showed no new lesions in the tonsils (**Supplementary Figure 1**). There was also no evidence of new metastasis or progression beyond the primary lesion during follow-up. Timeline of the relevant information is shown in **Table 2**.

**Table 2:** Timeline of the relevant information.

Time	Major medical examination	Diagnosis or efficacy evaluation	Treatment
2019.01.16–2019.01.29	Laryngoscopy; chest CT; cranial MRI; abdominal CT; whole body bone scan; NGS	Undifferentiated large-cell lung cancer of the left lung (T4NxM1b, AJCC 8^th^ edition)	Puncture of the lung lesions and tonsillectomy;
2019.02.15	-		Albumin-bound paclitaxel (200 mg on d 1 and 8) + carboplatin (450 mg on d 1);
2019.03.09	-		Albumin-bound paclitaxel (200 mg on d 1 and 8) + carboplatin (450 mg on d 1);
2019.04.02	-		Albumin-bound paclitaxel (200 mg on d 1 and 8) + carboplatin (450 mg on d 1);
2019.04.22	-		Albumin-bound paclitaxel (200 mg on d 1 and 8) + carboplatin (450 mg on d 1);
2019.05.15	Chest CT;	PD	
2019.05.22	-		Bronchial arterial infusion (cisplatin 40 mg + epirubicin 40 mg);
2019.06.21	Chest CT;	SD	Anlotinib;
2019.08.08	Chest CT;	SD	
2020.04.23	Chest CT;	SD	
2020.10.15	Chest CT;	SD	
2020.12.17	Chest CT;	SD	
2021.04.01	Chest CT;	SD	

All procedures performed in studies involving human participants were in accordance with the ethical standards of the institutional and/or national research committee(s) and with the Helsinki Declaration (as revised in 2013). Written informed consent was obtained from the patient for publication of this manuscript and any accompanying images.

## Discussion

The main pathological types of primary tonsil tumors are squamous cell carcinoma or lymphoma, and metastatic tumors of the tonsil are very rare (6). Metastatic tonsil tumors have been reported to account for only 0.8% of all tonsil tumors (7). Nearly 100 cases of metastatic malignant tumors of the tonsil have been reported, and the most common sources are digestive tract tumors, kidney cancer, and melanoma. In lung cancer, the primary pathological type of tonsil metastasis is small cell lung cancer, and this case is the first report of tonsil metastasis from undifferentiated large-cell lung cancer.

**Figure 3:**
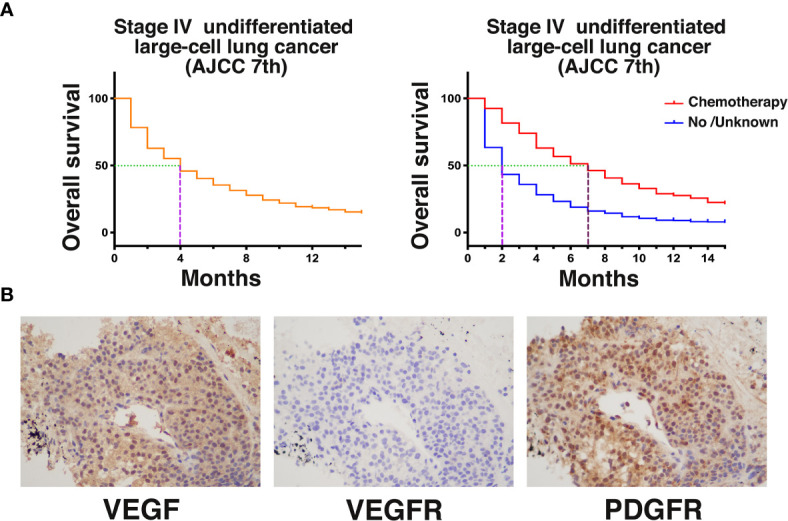
**(A)** Kaplan–Meier (KM) analysis of survival of patients with stage IV (AJCC 7^th^ edition) undifferentiated large-cell lung cancer from the SEER database after 2010. **(B)** Retrospective examination of the expression of angiogenesis-related genes and anlotinib targets by immunohistochemistry [VEGF (++); VEGFR (−); PDGFR (+++)].

Undifferentiated large-cell lung cancer is a type of NSCLC, but its incidence is significantly lower than that of common lung adenocarcinoma, lung squamous cell carcinoma, and other subtypes (8). The prognosis of patients with advanced undifferentiated large-cell lung cancer is poor (9). We obtained survival data from the Surveillance, Epidemiology, and End Results (SEER) database for 1,129 patients diagnosed with stage IV (AJCC 7^th^ edition) large-cell undifferentiated lung cancer after 2010 (**Figure 3A**). The results showed that the median OS was only 4 months. The median survival of those who received chemotherapy was only 5 months longer than that of those who did not or whose treatment status was unknown (7 *vs* 2 months, P < 0.0001). This report describes a case of stage IV undifferentiated large-cell lung cancer with rare tonsillar metastases for which genetic testing did not provide a therapeutic target. Platinum-based chemotherapy was selected as the first-line treatment. The PFS with chemotherapy was 3 months, which was similar to the database results.

The theory of cutting off the blood supply of tumors to combat them was first proposed in 1971. The first proangiogenesis factor, VEGF, was isolated in 1989. The main recognized tumor angiogenesis signaling pathways are the VEGF/VEGFR and PDGF/PDGFR pathways (10). At present, antiangiogenic therapy with bevacizumab is recommended for the treatment of lung cancer (3). However, there have been no clinical trials or case reports of antiangiogenic therapy for undifferentiated large-cell lung cancer. Anlotinib is an oral small-molecule multitarget antiangiogenic TKI, and its main targets include VEGFR, PDGFR, and other major angiogenesis-related factors. The Alter0303 study showed that the use of oral anlotinib monotherapy as third-line treatment for advanced NSCLC achieved a PFS of 4 months and an OS of 3.3 months (5). However, patients with large-cell undifferentiated carcinoma were not included in this study; therefore, the efficacy of anlotinib in patients with this type of lung cancer is unclear.

In this case, the tumor size rapidly increased after chemotherapy resistance occurred. We administered the oral antiangiogenic agent anlotinib. Early imaging during anlotinib treatment showed that the tumor remained stable and began to shrink slowly with prolonged treatment. At the last follow-up, the PFS and OS with anlotinib treatment were 21.5 and 27.5 months, respectively. There were no significant adverse reactions with this regimen. We retrospectively examined the expression of angiogenesis-related genes and anlotinib targets in this case by immunohistochemistry (**Figure 3B**). VEGF expression was positive, indicating that tumor angiogenesis activity was very high. Interestingly, VEGFR expression was negative. The expression of PDGFR, another important target of anlotinib, was positive, indicating that the PDGFR signaling pathway may be important for the function of anlotinib in the treatment of this patient. PDGFR is a tyrosine kinase receptor with two structurally related forms. Binding of PDGF and PDGFR activates downstream pathways that mediate angiogenesis (11). Studies have shown that PDGFR can be significantly overexpressed in lung tumors. Animal experiments have also shown that PDGFR inhibitors can significantly inhibit angiogenesis in tumors, while normal tissue angiogenesis is not affected (12). Therefore, the angiogenesis of PDGFR-positive vascular endothelial cells alone may not be significantly affected by PDGFR inhibitors. PDGFR immunohistochemistry in this case also indicated PDGFR positivity of the tumor. These results suggest that patients with PDGFR-positive tumors may receive additional benefit from PDGFR inhibitors such as anlotinib. This case suggests that for large-cell undifferentiated lung cancer patients who exhibit chemotherapy resistance, antiangiogenic therapy with anlotinib can be attempted and may have a good effect. PDGFR may be the target underlying this effect.

## Data Availability Statement

The original contributions presented in the study are included in the article/**Supplementary Material**. Further inquiries can be directed to the corresponding author.

## Ethics Statement

Ethical review and approval was not required for the study on human participants in accordance with the local legislation and institutional requirements. The patients/participants provided their written informed consent to participate in this study.

## Author Contributions

TX, CW, XZ, and BL collected the clinical information, diagnostic information, therapeutic information, and images of the patients. TX wrote the manuscript. ZW identified the case and submitted the manuscript. TX and XZ revised the manuscript. CW and ZW proofread the manuscript. TX, CW, XZ, and BL contributed equally to this work. All authors contributed to the article and approved the submitted version.

## Funding

This work was supported by grants from the Key Research and Development Plan (Social Development) of Science and Technology Department of Jiangsu Province (No. BE2019760); The Key young medical talents of Jiangsu Province (No. QNRC2016662); The “123” advantageous disciplines, core technologies and “789” excellent talent training plan of the Second Affiliated Hospital of Nanjing Medical University (No. 789ZYRC202090148).

## Conflict of Interest

The authors declare that the research was conducted in the absence of any commercial or financial relationships that could be construed as a potential conflict of interest.

## Supplementary Material

Supplementary Figure 1
**(A)** Cranial magnetic resonance imaging (MRI), abdominal CT, and whole body bone scan examinations showed no other metastatic lesions at initial diagnosis. **(B)** Metastatic lesion of tonsil before and after treatment.
